# Correlation of uric acid with microalbuminuria in Type-2 diabetic patients with normal creatinine

**DOI:** 10.12669/pjms.40.5.8208

**Published:** 2024

**Authors:** Saima Zeb, Bakht Babar, Samina Bibi, Muhammad Akbar Shah

**Affiliations:** 1 Dr. Saima Zeb, FCPS, MRCP, Department of Diabetes, Endocrinology and Metabolic Diseases, MTI Hayatabad Medical Complex, Peshawar, Pakistan; 2 Dr. Bakht Babar, Department of Diabetes, Endocrinology and Metabolic Diseases, MTI Hayatabad Medical Complex, Peshawar, Pakistan; 3 Dr. Samina Bibi, Department of Diabetes, Endocrinology and Metabolic Diseases, MTI Hayatabad Medical Complex, Peshawar, Pakistan; 4 Dr. Muhammad Akbar Shah, Department of Diabetes, Endocrinology and Metabolic Diseases, MTI Hayatabad Medical Complex, Peshawar, Pakistan

**Keywords:** Uric acid, Microalbuminuria, Type-2 diabetics, Creatinine, Diabetic kidney disease (DKD)

## Abstract

**Objective:**

To find the correlation of serum uric acid with microalbuminuria in Type-2 diabetic patients with normal creatinine.

**Methods:**

This cross-sectional study was conducted in the Department of Diabetes, Endocrinology and Metabolic diseases, Hayatabad Medical Complex, Peshawar, Pakistan from 1^st^ April, 2022 to 30^th^ September, 2022. Total 160 diabetic patients between the age of 30 and 65 years were enrolled in the study. Type-2 diabetic patients with microalbuminuria between 2.5 and 30 mg/mmol were included. The demographic details of patients were recorded in the questionnaire after taking consent. Fasting Uric acid, lipid profile and glucose along with creatinine and HbA1C were estimated from patient’s venous blood samples. Ratio of albumin to creatinine (ACR) in the random spot urine sample was used to detect microalbuminuria.

**Results:**

Out of 160 participants enrolled in the study there were 86 (54%) males and 74 (46%) females with the mean age of 50.15 ± 11.1 years and BMI of 20.93 kg/m^2^. Ninety six (60%) of the patients had Type-2 DM for less than five years, while remaining 64 (40%) were more than five years diabetic. Mean serum uric acid calculated was 6.85±2.06(mg/dl), while microalbuminuria was calculated as 8.02±0.78 (mg/mmol). The Pearson correlation of serum uric acid and microalbuminuria based on sex and age was statistically significant(p<0.05).

**Conclusion:**

We found that uric acid level was significantly associated with microalbuminuria in people with Type-2 diabetes with normal serum creatinine. Uric acid level can be a potential screening tool for early detection of DKD.

## INTRODUCTION

Uric acid is a byproduct of purine metabolism, around two-thirds of it is eliminated by the kidneys, while the remaining one-third is broken down in the intestines.^1^ Increase in serum uric acid is thought to be harmful to the kidneys due to reduced renal perfusion by increasing the proliferation of vascular smooth muscle cells in the afferent arteriolar tuft.^2^ Uric acid has been linked to kidney injury in diabetes individuals in studies that excluded those with high blood pressure as a contributing factor. Yet, increased blood insulin levels may impair the kidneys’ ability to excrete uric acid. It is generally agreed that hyperinsulinemia is central to the etiology of Type-2 diabetes. Albuminuria is also the major indicator of diabetic nephropathy in this population.^3^

Diabetes mellitus (DM) is a global epidemic. According to IDF 537 million people are already living with diabetes and by 2045 this number is projected to increase to 783 million.^4^ The cumulative occurrence of Type-2 DM in Pakistan is around 27%.^5^ Microalbuminuria is not only the hallmark of early detection of DKD but also an indication of endothelial dysfunction and therefore a risk factor for cardiovascular morbidity and mortality in diabetics.^6^ The end product of purine metabolism is uric acid, which is created by the enzyme xanthine oxidase. As a byproduct, xanthine oxidase also generates oxidants that have been linked to CVD and renal disease. As oxygen free radicals are produced during uric acid synthesis, this compound might serve as a convenient clinical indicator of oxidative stress.^7^ The routine screening of diabetic patients for hyperuricemia will help in identifying the group which is at higher risk of developing diabetes related complications.^6^

DKD is a slowly progressive disease and by the time it is clinically evident in the form of albuminuria and loss of renal function, significant parenchymal damage has already occurred. This highlights the importance of finding a new risk biomarker for early detection and thus prompt treatment of DKD.^8^ In Pakistan, limited research has been done on the link between microalbuminuria and uric acid in diabetic patients. Our research aimed to correlate uric acid with microalbuminuria in Type-2 diabetic patients with normal creatinine. This study will not only add to our knowledge regarding the possible use of uric acid for screening of early DKD, but also will be a gateway for further research work in this regard.

## METHODS

This cross-sectional study was conducted at the Department of Diabetes, Endocrinology and Metabolic diseases, Hayatabad Medical Complex, Peshawar, Pakistan between 1^st^ April 2022 and September 2022. Type-2 diabetic patients between the ages of 30 to 65years were included in the study. Patients with urinary tract infections, acute febrile illness, hepatic or renal diseases, gout, alcoholism, malignancy, chemotherapy, radiotherapy, pregnancy and menstruation were excluded.

### Ethical Approval

It was obtained from the institutions Ethical review committee (Ref. No. 591/HEC/B&PSC/2022, Dated 8^th^ March 2022).

Sample size was calculated on the WHO sample size calculator, a total of 160 subjects were chosen based on a prevalence of 27%^7^ Type-2 diabetes with 95% confidence interval and 5%. margin of error. The demographic details of patients including age, gender, height and weight for BMI, smoking details, duration of disease were recorded in the questionnaire after taking consent. Fasting Uric acid, lipid profile and glucose along with creatinine and HbA1C were estimated from venous blood samples. Regarding diet all the patients were non-vegetarian with no protein restriction. Ratio of albumin to creatinine (ACR) in random spot urine sample was used to detect microalbuminuria.

The data analysis for this study was carried out using version 26.0 of the IBM-SPSS. Descriptive analysis was performed on demographic factors such as age, gender, BMI etc. Independent t-test and Pearson’s correlation were used to determine the mean and standard deviation of biochemical parameters. If the p-value was lower than 0.05, the data were considered to be statistically significant.

## RESULTS

Out of 160 participants, 86 (54%) were males and 74 (46%) were females. Mean age was 50.15 ± 11.1 years and BMI of 20.93 kg/m^2^ Out of these 160 participants 96 (60%) had Type-2 DM for less than five years, however, 64 (40%) had diabetes for more than five years. Mean serum uric acid calculated was 6.85±2.06 (mg/dl), while microalbuminuria was calculated as 8.02±0.78 (mg/mmol), (Table I).

**Table-I:** Demographic details of the patient data.

Variables	N	%
Male	86	54
Female	74	46
Smoking	22	13.8
No smoking	138	86.3

*Variables*	*Mean*	*Std. Deviation*

Age (years)	50.15	11.12
BMI (kg/m^2^)	20.93	5.22
Duration of disease	5.35	1.76
HbA1C (%)	11.19	2.45
Serum creatinine (mg/dl)	.75	.29
Mean serum uric acid	6.85	2.06
Serum uric acid (3.5 – 7.5mg/dl) (Male)	7.1	1.26
Serum uric acid (2.6 – 6.5mg/dl) (Female)	6.4	2.45
Microalbuminuria (mg/mmol)	8.02	0.78
Systolic BP (mmHg)	106.27	12.65
Diastolic BP (mmHg)	74.07	11.33
Total cholesterol (mg/dl)	178.21	35.27
TG (mg/dl)	184.66	102.79
HDL (mg/dl)	45.01	11.25
LDL (mg/dl)	110.45	26.46
Glucose (mg/dl)	109.67	56.49

The patient data were divided in to two groups, on the basis of duration of diabetes i.e., less than five years and more than five years. Table-II BMI, age, HBA1C and serum uric acid were significantly increased in the patients who had diabetes for more than five years. However, creatinine, microalbuminuria, blood pressure, lipid profile and glucose were not significant.

**Table-II:** Comparison of patients with more and less five years of diabetes.

Grouping based on duration of disease		Mean	Std. Deviation	p-Value
BMI (kg/m2)	≤5	20.8	5.3	0.045*
	>5	21.7	4.8	
Age (years)	≤5	50.0	10.8	0.032*
	>5	52.7	12.4	
HbA1C (%)	≤5	11.1	2.5	0.049*
	>5	13.7	2.4	
Serum creatinine (mg/dl)	≤5	0.8	0.3	0.645
	>5	0.9	0.3	
Serum uric acid (mg/dl) (Male)	≤5	6.7	2.1	0.042*
	>5	7.2	1.92	
Serum uric acid (mg/dl) (Female)	≤5	6.3	2.4	0.048^*^
	>5	6.6	2.0	
Microalbuminuria (mg/mmol)	≤5	6.2	0.8	0.052
	>5	7.6	0.7	
Systolic BP (mmHg)	≤5	105.8	12.4	0.353
	>5	108.0	13.7	
Diastolic BP(mmHg)	≤5	74.2	11.3	0.615
	>5	73.6	11.7	
Total Cholestrol (mg/dl)	≤5	178.0	35.4	0.83
	>5	179.2	35.4	
TG (mg/dl)	≤5	188.3	103.7	0.679
	>5	170.3	99.5	
HDL (mg/dl)	≤5	44.6	11.5	0.791
	>5	46.5	10.0	
LDL (mg/dl)	≤5	110.6	26.8	0.674
	>5	109.9	25.5	
Glucose (mg/dl)	≤5	111.5	60.4	0.177
	>5	102.4	36.8	

The Pearson correlation of serum uric acid and micro albuminuria is shown in Table-III. The data was stratified based on sex, age and duration of Type-2 diabetes. For control variables of sex and age the significant positive correlation between serum uric acid and microalbuminuria was observed.

**Table-III:** Correlation analysis of Hyperuricaemia and micro albuminuria of diabetic patients.

Control Variables	Correlation	Person’s Test	

		r2	P-value
Sex	Microalbuminuria (mg/mmol) x Serum uric acid (mg/dl)	0.076	0.045*
Age	Microalbuminuria (mg/mmol) x Serum uric acid (mg/dl)	0.095	0.036*
Duration of disease	Microalbuminuria (mg/mmol) x Serum uric acid (mg/dl)	0.089	0.62

## DISCUSSION

Increasing evidence suggests that serum uric acid, an inflammatory agent contributes significantly to endothelial dysfunction.^9^ As a result of these observations, we evaluated the correlation of uric acid with microalbuminuria in Type-2 diabetics patients with normal creatinine. One hundred and sixty people with Type-2 diabetes participated in our research. The r-value of 0.076 and 0.095 indicates a positive relationship between the serum uric acid level and microalbuminuria level with controlled variables of sex and age respectively. Serum uric acid was shown to have a significant positive link with diabetic nephropathy in research by Xia Q et al. It was shown that the blood uric acid level significantly contributed to the development of nephropathy in patients with Type-2 diabetes.^10^

Researchers Du J et al. and coworkers found a statistically significant correlation between serum uric acid levels and urinary albumin excretion (UAE). Age, time from diagnosis, and length of diabetes all showed positive correlations. Serum uric acid concentration, systolic blood pressure, hemoglobin A1c, and total duration of DM were all shown to be significant predictors of UAE in a multiple regression analysis.^11^ Similarly, Warjukar et al. found a positive correlation between urine microalbuminuria and serum uric acid levels (p=0.001), drawing the conclusion that both serum uric acid and urine microalbumin levels can serve as early diagnostic markers for kidney and atherogenic cardiovascular diseases. These findings are also useful for defining the prognostic monitoring of the disease in Type-2 diabetics. It is also common for people with chronic renal failure to have hyperuricemia. Several investigations have indicated that hyperuricemia may have a pathogenic role in the onset and progression of chronic renal failure, rather than only demonstrating impaired uric acid excretion by the kidneys. Elevated serum uric acid in diabetic individuals is strongly linked with the subsequent onset of chronic macroalbuminuria.^12^

In this study BMI and HbA1C both are significantly increased in those patients who had disease for more than five years. Nevertheless, our results showed that the concentration of serum uric acid was much greater in those who had metabolic syndrome. Hyperuricemia has been linked to insulin resistance and the development or worsening of nephropathy in people with Type-2 diabetes, according to research by Johnson RJ et al.^13^ Obesity, high blood sugar, high insulin levels, high triglyceride levels, and high blood pressure all contribute to the development of metabolic syndrome and early cardiovascular changes.^14^ Foster et al showed that serum triglyceride, fasting blood glucose, and hemoglobin A1c levels were all significantly correlated with hyperuricemia.^15^ But in our study the correlation of blood pressure with the duration of the disease was insignificant. It’s important to note that our research used the criteria of a blood pressure reading of at least 140/90 mm Hg or the use of antihypertensive medication to identify participants as having hypertension, similarly observed by Al Mansour and Mohammed Abdullah.^16^

Consequently, if hyperuricemia is identified and treated in diabetic patients at an early stage, renal failure may be avoided. Allopurinol, a xanthine oxidase inhibitor, is effective in reducing uric acid levels. Allopurinol, when taken at the maximum dose of 600 milligrams per day, may reduce blood uric acid levels by as much as sixty percent, while a reduction of thirty to forty percent can be obtained at the recommended dosage of three hundred milligrams per day.^17^ Gherghina et al., conducted research demonstrating that allopurinol’s ability to decrease uric acid levels can reverse many of uric acid’s negative effects, including suppression of the renin angiotensin aldosterone system, decreased oxidative stress, improved nitric oxide bioavailability, improved endothelial function, and decreased concentrations of biomarkers for urinary inflammation.^18^ So, early treatment of hyperuricemia in Type-2 diabetic may help to prevent the progression to renal failure.

### Limitations

This is a single center study and because of the poor follow up of the patient it was impossible to do longitudinal studies. However, in future keeping in mind the finding of this study we are planning to have longitudinal, multicenter study with possible intervention of xanthine oxidase inhibitor which can help overcome these limitations.

## CONCLUSION

This study found a strong relationship between microalbuminuria and serum uric acid in people with Type-2 diabetes, Serum uric acid can be used as an early diagnostic marker for diabetic nephropathy. Laboratory measurement of uric acid is simple, fast and non expensive Hyperuricemia treatment is also easily accessible, thus identifying and treating it early may aid in slowing the progression of renal disease. Our results are preliminary and further research is needed to confirm them.

### Authors Contribution:

**SZ:** Concept and design of the study, Principal investigator, interpretation of data, Reviewed the manuscript, Final approval.

**BB:** Principal investigator, designing the study, data collection and compilation, manuscript writing, statistical analysis, Literature search, supervision, final approval and responsible for the accuracy study.

**SB:** Data collection, Literature search, Data interpretation, Drafting, Critical revision of article.

**MAS:** Data collection, Data analysis and interpretation, Literature search, Drafting, Critical revision.

## References

[1] Xie WR, Yang XY, Deng ZH, Zheng YM, Zhang R, Wu LH (2022). Effects of washed microbiota transplantation on serum uric acid levels, symptoms, and intestinal barrier function in patients with acute and recurrent gout:A pilot study. Digestive Dis.

[2] Russo E, Verzola D, Cappadona F, Leoncini G, Garibotto G, Pontremoli R (2021). The role of uric acid in renal damage-a history of inflammatory pathways and vascular remodeling. Vessel Plus.

[3] Li CH, Lee CL, Hsieh YC, Chen CH, Wu J, Tsai SF (2022). Hyperuricemia and diabetes mellitus when occurred together have higher risks than alone on all-cause mortality and end-stage renal disease in patients with chronic kidney disease. BMC Nephrol.

[4] Magliano DJ, Boyok EJ (2021). IDF Diabetes Atlas 10^th^ edition scientfic committee. IDF DIABETES ATLAS.

[5] Zafar J, Nadeem D, Khan SA, Jawad Abbasi MM, Aziz F, Saeed S (2016). Prevalence of diabetes and its correlates in urban population of Pakistan:A Cross-sectional survey. J Pak Med Assoc.

[6] Jalal DI, Rivard CJ, Johnson RJ, Maahs DM, McFann K, Rewers M (2010). Serum uric acid levels predict the development of albuminuria over 6?years in patients with type 1 diabetes:Findings from the Coronary Artery Calcification in Type 1 Diabetes study. Nephrol Dial Transplantat.

[7] Liu N, Xu H, Sun Q, Yu X, Chen W, Wei H (2021). The role of oxidative stress in hyperuricemia and xanthine oxidoreductase (XOR) inhibitors. Oxid Med Cell Longev.

[8] Rajkarnikar P, Xu Y, Bhattarai A (2020). Effects of Hyperuricemia on Beta-Cell Function, Renal Function, and Lipid Panels of Patients with Diabetic Kidney Disease:A Gender-Based Retrospective Study. Dubai Diabetes Endocrinol J.

[9] Haryono A, Nugrahaningsih DAA, Sari DCR, Romi MM, Arfian N (2018). Reduction of serum uric acid associated with attenuation of renal injury, inflammation and macrophages M1/M2 ratio in hyperuricemic mice model. Kobe J Med Sci.

[10] Xia Q, Zhang S-H, Yang S-M, Zhu X-L, Su S, Hu A-P (2020). Serum uric acid is independently associated with diabetic nephropathy but not diabetic retinopathy in patients with type 2 diabetes mellitus. J Chinese Med Assoc.

[11] Du J, Xu J, Wang X, Liu Y, Zhao X, Zhang H (2020). Reduced serum CTRP12 levels in type 2 diabetes are associated with renal dysfunction. Int Urol Nephrol.

[12] Warjukar P, Jain P, Kute P, Anjankar A, Ghangale S (2020). Study of microalbuminuria and uric acid in type 2 diabetes mellitus. Int J Cur Res Rev.

[13] Johnson RJ, Bakris GL, Borghi C, Chonchol MB, Feldman D, Lanaspa MA (2018). Hyperuricemia, acute and chronic kidney disease, hypertension, and cardiovascular disease:report of a scientific workshop organized by the National Kidney Foundation. Am J Kidney Dis.

[14] Manzoor M, Babar B (2023). Left ventricular diastolic dysfunction and its pattern in type 2 diabetic patients with and without microalbuminuria. J Postgrad Med Instit.

[15] Foster C, Smith L, Alemzadeh R (2020). Excess serum uric acid is associated with metabolic syndrome in obese adolescent patients. J Diabet Metabolic Disord.

[16] Al Mansour MA (2020). The prevalence and risk factors of type 2 diabetes mellitus (DMT2) in a semi-urban Saudi population. Int J Environ Res Public Health.

[17] Chu WY, Annink KV, Nijstad AL, Maiwald CA, Schroth M, Bakkali Le (2022). Pharmacokinetic/pharmacodynamic modelling of allopurinol, its active metabolite oxypurinol, and biomarkers hypoxanthine, xanthine and uric acid in hypoxic-ischemic encephalopathy neonates. Clin Pharmacokinet.

[18] Gherghina ME, Peride I, Tiglis M, Neagu TP, Niculae A, Checherita IA (2022). Uric Acid and Oxidative Stress—Relationship with Cardiovascular, Metabolic, and Renal Impairment. Int J Mol Sci.

